# ESR1 Gene Polymorphisms and Prostate Cancer Risk: A HuGE Review and Meta-Analysis

**DOI:** 10.1371/journal.pone.0066999

**Published:** 2013-06-21

**Authors:** Yu-Mei Wang, Zu-Wang Liu, Jing-Bo Guo, Xiao-Fang Wang, Xin-Xin Zhao, Xuan Zheng

**Affiliations:** Department of Hospice, Shengjing Hospital of China Medical University, Shenyang, China; University of Texas Health Science Center, United States of America

## Abstract

**Background:**

Many published data on the association between single nucleotide polymorphisms (SNPs) in the ESR1 gene and prostate cancer susceptibility are inconclusive. The aim of this Human Genome Epidemiology (HuGE) review and meta-analysis is to derive a more precise estimation of this relationship.

**Methods:**

A literature search of PubMed, Embase, Web of Science and Chinese Biomedical (CBM) databases was conducted from their inception through July 1st, 2012. Crude odds ratios (ORs) with 95% confidence intervals (CIs) were calculated to assess the strength of association.

**Results:**

Twelve case-control studies were included with a total 2,165 prostate cancer cases and 3,361 healthy controls. When all the eligible studies were pooled into the meta-analysis, ESR1 PvuII (C>T) and XbaI (A>G) polymorphisms showed no association with the risk of prostate cancer. However, in the stratified analyses based on ethnicity and country, the results indicated that ESR1 PvuII (C>T) polymorphism was significantly associated with increased risk of prostate cancer among Asian populations, especially among Indian population; while ESR1 XbaI (A>G) polymorphism may significantly increase the risk of prostate cancer among American population. Furthermore, we also performed a pooled analysis for all eligible case-control studies to explore the role of codon 10 (T>C), codon 325 (C>G), codon 594 (G>A) and +261G>C polymorphisms in prostate cancer risk. Nevertheless, no significant associations between these polymorphisms and the risk of prostate cancer were observed.

**Conclusion:**

Results from the current meta-analysis indicate that ESR1 PvuII (C>T) polymorphism may be a risk factor for prostate cancer among Asian populations, especially among Indian population; while ESR1 XbaI (A>G) polymorphism may increase the risk of prostate cancer among American population.

## Introduction

Prostate cancer is the second most frequently diagnosed cancer and the sixth leading cause of cancer deaths in males. It accounted for 14% (903,500) of the total new cancer cases and 6% (258,400) of the total cancer deaths in males in 2008 [1]. Generally, prostate cancer is known to be a multifactorial disease induced by complex interactions between environmental and genetic factors [2]. Hormonal factors also play a fundamental role in the progression of prostate cancer through estrogen synthesis, metabolism and signal transduction pathways [3]. In the last decade, evidences point to genetic factors, such as variations in hormonal gene, as the key players in prostate cancer development. Currently, a wide range of genes have been identified have some risk associations with prostate cancer, such as AR, CYP17/19, NOS, PSA, ESR1/2, etc [4]–[12].

Estrogen receptor 1 (ESR1) is located on chromosome 6, locus 6p25.1 and spans approximately 300 kb in length, including 8 exons and 7 introns [13]. ESR1 functions as a ligand-activated transcription factor composed of several domains important for hormone binding, DNA binding, as well as activation of transcriptions; it can also interact with estrogens receptors to stimulate proliferation of mammary epithelial tissue and alter the expression of downstream genes [14]. Generally, ESR1 is implicated in prostate cancer susceptibility by stimulating aberrant prostate growth, controlling prostate cell growth and programming prostate cell death [15]. Recently, several ESR1 gene polymorphisms have been identified as candidates for prostate cancer susceptibility and among these, ESR1 PvuII (rs2234693 C>T) and XbaI (rs9340799 A>G) polymorphisms were suggested to possess significant associations with the development of prostate cancer. Both PvuII and XbaI can affect ESR1 transcription activity and possibly contribute to the elevated risk of prostate cancer [3], [6], [16], but the exact effects of ESR1 gene mutations on prostate epithelial cells are still debated despite the fact that estrogen is already used in treatming prostate cancer due to its growth-inhibitory effects [17]. A recent case-control study observed no associations between the selected genetic polymorphisms of ESR1 and prostate cancer risk [14]. Sun et al also suggested that common genetic variations in ESR1 did not strongly correlate with prostate cancer aggressiveness and they also indicated that the polymorphisms of ESR1 may have no biological functions [5]. The inconsistent conclusions to link ESR1 gene mutations with the risk of prostate cancer may be due to the limitations in sample size in the corresponding investigations, of in the inadequate statistical power in genetic studies of complex traits, like age, ethnicity, gender, the histological type, differentiation on tumor stage and research methodology [16]. Therefore, we performed a meta-analysis of all eligible case-control studies with prostate cancer risk and aimed to reveal a more precise relationship between ESR1 gene polymorphisms and prostate cancer susceptibility. Such relationship will shed light on a comprehensive functional profiling of ESR1 gene for better understanding of the biological processes associated with prostate cancer formation and progression [17]. Furthermore, identification of common polymorphisms in the ESR1 gene may be useful in early diagnosis of prostate cancer, allowing patients to receive timely and effective anti-cancer therapies.

## Materials and Methods

### Literature search

Relevant papers published before July 1^th^, 2012 were identified through a literature search in PubMed, Embase, Web of Science and Chinese Biomedical (CBM) databases using the following terms: (″genetic polymorphism″ or ″polymorphism″ or ″SNP″ or ″single nucleotide polymorphism″ or ″gene mutation″ or ″genetic variants″) and (″prostatic neoplasms″ or ″prostate neoplasm″ or ″prostate cancer″ or ″prostatic cancer″) and (″estrogen receptor alpha″ or ″estradiol receptor alpha″ or ″ER alpha″ or ″Estrogen Receptor 1″ or ″ESR1″). The references from the eligible articles or textbooks were also manually searched to find other potential studies. Disagreements were resolved through discussions between the authors.

### Inclusion and Exclusion Criteria

Studies included in our meta-analysis have to meet the following criteria: (a) case-control studies or cohort studies focused on associations between ESR1 gene polymorphisms and prostate cancer susceptibility; (b) all patients diagnosed with prostate cancer should be confirmed by pathological or histological examinations; (c) published data about the frequencies of alleles or genotypes must be sufficient; (d) studies were published in English or Chinese. Studies were excluded when they were: (a) not a case-control study or a cohort study; (b) duplicates of previous publications; (c) based on incomplete data; (d) meta-analyses, letters, reviews or editorial articles. If more than one study by the same author using the same case series were published, either the studies with the largest sample size or the most recently published study was included. The supporting PRISMA checklist is available as supporting information; see Supplement S1.

### Data Extraction

Using a standardized form, data from published studies were extracted independently by two authors. The following information were extracted from each article: the first author, year of publication, country, language, ethnicity, study design, numbers of subjects, source of cases and controls, detecting sample, genotype method, allele and genotype frequencies, and evidence of Hardy-Weinberg equilibrium (HWE) in controls. An attempt was made to contact authors if data presentation was incomplete or if it was necessary to resolve an apparent conflict or inconsistency in the article. In case of conflicting evaluations, disagreements were resolved through discussion between the authors.

### Quality assessment of included studies

Two authors independently assessed the quality of papers according to the modified STROBE quality score systems [18]. Forty assessment items related to the quality appraisal were used in this meta-analysis with scores ranging from 0 to 40. Scores of 0–20, 20–30 and 30–40 were defined as low, moderate and high quality, respectively. Disagreements were also resolved through discussion between the authors. The supporting modified STROBE quality score systems is available in Supplement S2. The methodological quality of all eligible studies was also evaluated using the Newcastle-Ottawa Scale (NOS) [19]. The NOS criteria uses a “star” rating system to judge methodological quality, which was based on three perspectives of the study: selection, comparability, and exposure. Scores, ranged from 0 stars (worst) to 9 stars (best), equal to or higher than 7 indicated that the methodological quality was generally good. The supporting NOS quality assessment scale is available in Supplement S3.

### Statistical Analysis

The association strength between ESR1 gene polymorphisms and prostate cancer susceptibility was measured by odds ratios (ORs) and 95% confidence intervals (CIs) under five genetic models: allele model (mutant [M] allele versus wild [W] allele), dominant model (WM+MM versus WW), recessive model (MM versus WW+WM), homozygous model (MM versus WW), and heterozygous model (MM versus WM). The statistical significance of the pooled OR was examined using the Z test. Between-study heterogeneities were estimated using Cochran's Q-statistic with a *P*<0.05 as statistically significant heterogeneity [20]. We also quantified the effect of heterogeneity using the *I^2^* test (ranged from 0 to 100%), which represents the proportion of inter-study variability that can be contributed to heterogeneity rather than to chance [21]. When a significant Q-test has *P*<0.05 or *I^2^* > 50%, it indicates that heterogeneity among studies existed and the random effects model (DerSimonian Laird method) was conducted for meta-analysis; otherwise, the fixed effects model (Mantel-Haenszel method) was used. To establish the effects of heterogeneity based on the results from the meta-analyses, we also performed subgroup analysis by ethnicity, country, source of controls, and genotype methods. We tested whether genotype frequencies of controls were in HWE using the χ^2^ test. Sensitivity analysis was performed through omitting each study in order to assess the quality and consistency of the results. Begger's funnel plots and Egger's linear regression test were used to evaluate publication bias [22]. All tests were two-sided and a P value of<0.05 was considered statistically significant. All analyses were calculated using the STATA software, version 12.0 (Stata Corp, College Station, TX, USA).

## Results

### The characteristics of included studies

**Figure 1:**
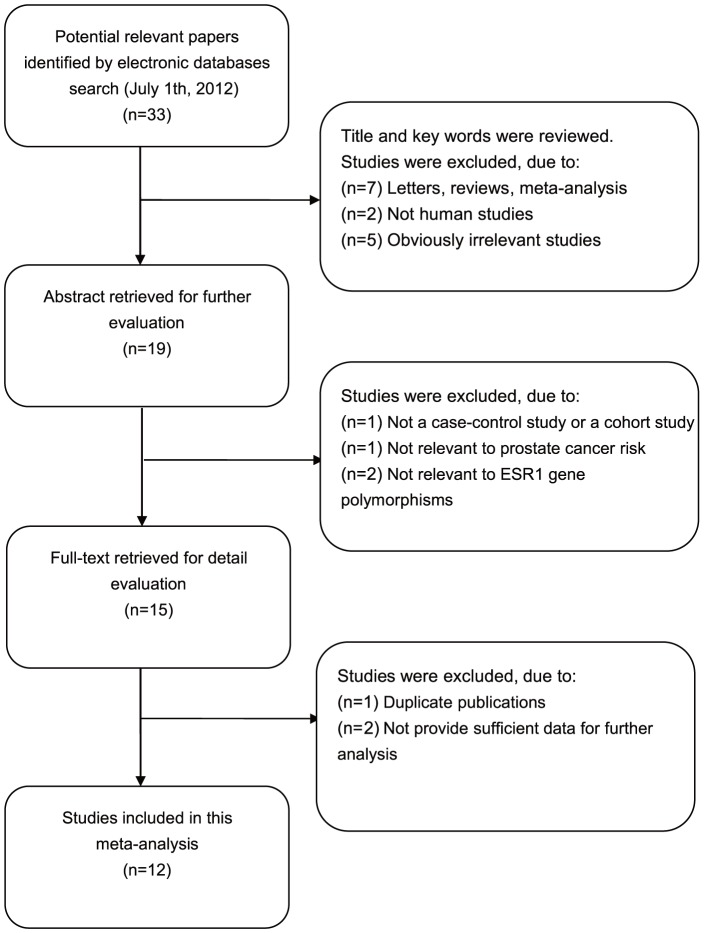
Flow chart of literature search and study selection.

According to the inclusion criteria, 12 case-control studies [4], [12], [23]–[32] were included were excluded in this meta-analysis. The flow chart that displays the study selection process is shown in Figure 1. A total of 2,165 prostate cancer cases and 3,361 controls were included in this meta-analysis. The publication year of involved studies ranged from 2001 to 2011. All patients diagnosed with prostate cancer were also confirmed by histopathological examinations. Four studies used hospital-based controls, while the other eight studies used population-based controls (community populations). Among these studies, four studies were performed in Caucasian populations, seven studies in Asian populations and one study in mixed populations. Tissue samples were used for genotyping in three studies, while the rest used blood samples for genotyping. Various genotype methods were used among these studies, including polymerase chain reaction-single strand conformation polymorphism (PCR-SSCP), denaturing high performance liquid chromatography (DHPLC), direct DNA sequencing, Taqman, and PCR-restriction fragment length polymorphism (PCR-RFLP). Six single nucleotide polymorphisms (SNPs) in the ESR gene were considered, including PvuII (rs2234693 C>T), XbaI (rs9340799 A>G), codon 10 (rs2077647 T>C), codon 325 (rs1801132 C>G), codon 594 (rs2228480 G>A) and +261G>C (rs746432 G>C); and among these, PvuII (C>T) and XbaI (A>G) polymorphism were the most common SNPs. Genotype frequencies among the controls were consistent with the Hardy-Weinberg equilibrium (HWE) test, except for four studies [25], [29], [31], [32]. The characteristics and methodological quality of the included studies are summarized in Table 1.

**Table 1:** Characteristics of included studies in this meta-analysis.

First author [Ref]	Year	Country	Ethnicity	Number		Source		Sample	Genotype method	SNP ID	Alternate name	STROBE score	NOS scale
				Case	Control	Case	Control						
Modugno et al [29]	2001	USA	Caucasian	88	241	HB	PB	Blood	PCR-SSCP	rs9340799 (A>G)	XbaI	26	5/9
										rs2234693 (C>T)	PvuII		
Cancel-Tassin et al [25]	2003	France	Caucasian	96	96	HB	PB	Blood	DHPLC	rs2077647 (T>C)	Codon 10	25	7/9
										rs746432 (G>C)	+261G>C		
										rs1801132 (C>G)	Codon 325		
										rs2228480 (G>A)	Codon 594		
Suzuki et al [33]	2003	Japan	Asian	101	114	HB	HB	Blood	PCR-SSCP	rs2234693 (C>T)	PvuII	26	6/9
										rs9340799 (A>G)	XbaI		
Tanaka et al [34]	2003	USA	Asian	115	200	HB	PB	Tissue	DNA sequencing	rs2077647 (T>C)	Codon 10	26	7/9
										rs2234693 (C>T)	PvuII		
										rs1801132 (C>G)	Codon 325		
										rs2228480 (G>A)	Codon 594		
Fukatsu et al [26]	2004	Japan	Asian	147	266	HB	HB	Tissue	PCR-SSCP	rs9340799 (A>G)	XbaI	27	8/9
										rs2234693 (C>T)	PvuII		
Hernandez et al [28]	2006	USA	Mixed	598	1098	HB	HB	Blood	TaqMan	rs9340799 (A>G)	XbaI	26	6/9
										rs2234693 (C>T)	PvuII		
Onsory et al [6]	2008	India	Asian	100	100	HB	HB	Tissue	PCR-RFLP	rs2234693 (C>T)	PvuII	23	6/9
Sobti et al [31]	2008	India	Asian	157	170	HB	PB	Blood	PCR-RFLP	rs2234693 (C>T)	PvuII	24	5/9
Chae et al [16]	2009	USA	Caucasian	269	440	HB	PB	Blood	TaqMan	rs1801132 (C>G)	Codon 325	26	7/9
										rs2077647 (T>C)	Codon 10		
										rs746432 (G>C)	+261G>C		
										rs2228480 (G>A)	Codon 594		
Gupta et al [27]	2010	India	Asian	157	170	HB	PB	Blood	PCR-RFLP	rs2234693 (C>T)	PvuII	24	8/9
										rs9340799 (A>G)	XbaI		
Sonoda et al [32]	2010	Japan	Asian	180	177	PB	PB	Blood	TaqMan	rs2077647 (T>C)	Codon 10	25	8/9
										rs2234693 (C>T)	PvuII		
										rs1801132 (C>G)	Codon 325		
Sissung et al [30]	2011	USA	Caucasian	157	289	HB	PB	Blood	PCR-RFLP	rs2234693 (C>T)	PvuII	24	9/9
										rs9340799 (A>G)	XbaI		

Ref  =  reference; PCR-RFLP  =  polymerase chain reaction-restriction fragment length polymorphism; PCR-SSCP  =  polymerase chain reaction-single strand conformation polymorphism; DHPLC  =  denaturing high performance liquid chromatography; HB  =  Hospital-based; PB  =  Population-based; NOS  =  Newcastle-Ottawa Scale.

### Association between ESR1 PvuII (C>T) polymorphism and prostate cancer risk

**Figure 2:**
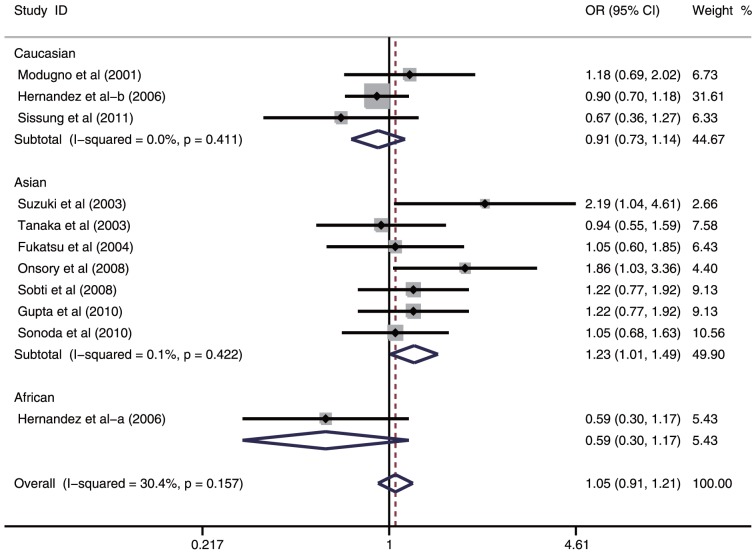
Subgroup analysis by ethnicity of ORs with a random-effects model for associations between ESR1 PvuII (C>T) polymorphism and prostate cancer risk under dominant model (TT + TC vs. CC).

**Figure 3:**
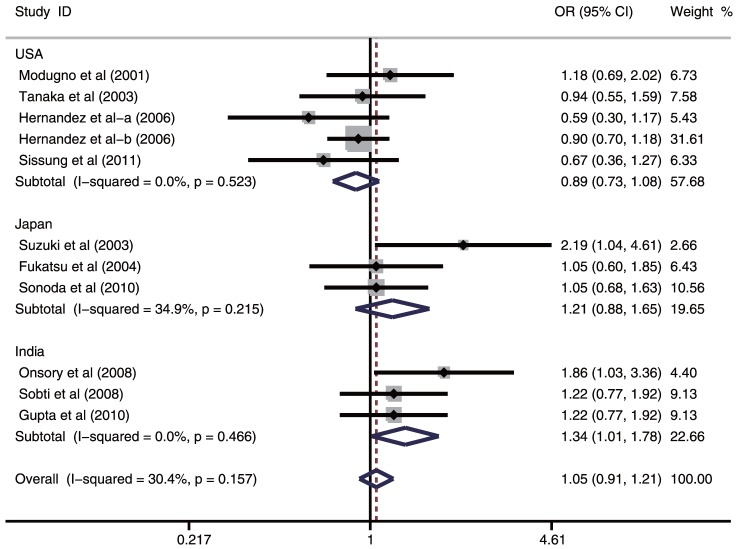
Subgroup analysis by country of ORs with a random-effects model for associations between ESR1 PvuII (C>T) polymorphism and prostate cancer risk under dominant model (TT + TC vs. CC).

A summary of the meta-analysis findings on the association between ESR1 PvuII (C>T) and prostate cancer risk is provided in Table 2. Ten studies involved the correlations between ESR1 PvuII (C>T) polymorphism and prostate cancer risk. The heterogeneity obviously existed under four genetic models (all *P*<0.05), which might be a result of the difference in ethnicity, country, source of controls and genotype methods, so random effects model was conducted to pool the results. The meta-analysis results showed that ESR1 PvuII (C>T) polymorphism is not linked to the risk of prostate cancer under all genetic models (T allele vs. C allele: OR  =  1.10, 95%CI: 0.91–1.33, *P* = 0.332; TT + TC vs. CC: OR = 1.05, 95%CI: 0.91–1.21, *P* = 0.478; TT vs. CC + CT: OR = 1.21, 95%CI: 0.87–1.69, *P* = 0.255; TT vs. CC: OR = 1.26, 95%CI: 0.85–1.86, *P* = 0.256; TT vs. CT: OR = 1.19, 95%CI: 0.87–1.61, *P* = 0.277; respectively). In the stratified analysis by ethnicity, ESR1 PvuII (C>T) is significantly correlated with increased risk of prostate cancer among Asian populations (T allele vs. C allele: OR = 1.28, 95%CI: 1.05–1.57, *P* = 0.015; TT + TC vs. CC: OR = 1.23, 95%CI: 1.01–1.49, *P* = 0.039; TT vs. CC + CT: OR = 1.59, 95%CI: 1.10–2.30; *P* = 0.016; TT vs. CC: OR = 1.77, 95%CI: 1.16–2.72, *P* = 0.009; TT vs. CT: OR = 1.49, 95%CI: 1.06–2.09, *P* = 0.023; respectively) (Figure 2). However, similar associations were not observed among Caucasian and African populations (all *P* > 0.05). Further subgroup analysis based on country suggested that ESR1 PvuII (C>T) may be associated with increased risk of prostate cancer among Indian population (T allele vs. C allele: OR = 1.37, 95%CI: 1.13–1.67, *P* = 0.001; TT + TC vs. CC: OR = 1.34, 95%CI: 1.01–1.78, *P* = 0.040; TT vs. CC + CT: OR = 2.06, 95%CI: 1.37–3.09, *P*<0.001; TT vs. CC: OR = 2.27, 95%CI: 1.46–3.53, *P*<0.001; TT vs. CT: OR = 1.93, 95%CI: 1.26–2.94, *P* = 0.002; respectively) (Figure 3), but similar results were not found among American or Japanese populations (all *P*>0.05). Subgroup analyses based on source of controls and genotype methods, we also found no correlations between ESR1 PvuII (C>T) and the risk of prostate cancer (all *P*>0.05) (shown in Table 2).

**Table 2:** Meta-analysis of the association between ESR1 PvuII (C>T) polymorphism and prostate cancer risk.

Subgroups (sample sizes)	T allele vs. C allele (allele model)				TT + TC vs. CC (dominant model)				TT vs. CC + CT (recessive model)				TT vs. CC (homozygous model)				TT vs. CT (heterozygous model)			
	OR	95%CI	*P*	*P_h_*	OR	95%CI	*P*	*P_h_*	OR	95%CI	*P*	*P_h_*	OR	95%CI	*P*	*P_h_*	OR	95%CI	*P*	*P_h_*
Overall (n = 11)	1.10	0.91–1.33	0.332†	<0.001	1.05	0.91–1.21	0.478	0.157	1.21	0.87–1.69	0.255†	<0.001	1.26	0.85–1.86	0.256†	<0.001	1.19	0.87–1.61	0.277†	0.002
*Ethnicity*																				
Caucasians (n = 3)	0.90	0.66–1.24	0.518†	0.023	0.91	0.73–1.14	0.422	0.411	0.84	0.46–1.52	0.562†	0.006	0.83	0.44–1.56	0.552†	0.024	0.85	0.47–1.51	0.573†	0.014
Asians (n = 7)	1.28	1.05–1.57	0.015†	0.062	1.23	1.01–1.49	0.039	0.422	1.59	1.10–2.30	0.016†	0.049	1.77	1.16–2.72	0.009†	0.075	1.49	1.06–2.09	0.023	0.131
Africans (n = 1)	0.74	0.47–1.16	0.192	-	0.59	0.30–1.18	0.135	-	0.77	0.35–1.71	0.522	-	0.56	0.23–1.39	0.213	-	0.93	0.40–2.15	0.855	-
*Country*																				
American (n = 5)	0.89	0.73–1.09	0.274†	0.073	0.89	0.73–1.08	0.227	0.523	0.86	0.60–1.25	0.438†	0.033	0.81	0.54–1.22	0.318†	0.078	0.89	0.62–1.28	0.537†	0.065
Japanese (n = 3)	1.35	0.70–2.62	0.374†	0.009	1.21	0.88–1.65	0.239	0.215	1.47	0.56–3.89	0.437†	0.009	1.78	0.52–6.10	0.359†	0.018	1.36	0.56–3.30	0.494†	0.025
Indian (n = 3)	1.37	1.13–1.67	0.001	0.721	1.34	1.01–1.78	0.040	0.466	2.06	1.37–3.09	<0.001	0.993	2.27	1.46–3.53	<0.001	0.913	1.93	1.26–2.94	0.002	0.889
*Source of controls*																				
Population-based (n = 5)	1.07	0.82–1.40	0.639†	0.011	1.07	0.87–1.30	0.539	0.693	1.23	0.67–2.24	0.501†	0.001	1.24	0.67–2.28	0.492†	0.005	1.23	0.68–2.20	0.492†	0.002
Hospital-based (n = 6)	1.14	0.84–1.54	0.413†	0.001	1.04	0.85–1.27	0.696	0.024	1.20	0.78–1.84	0.408†	0.009	1.28	0.71–2.31	0.410†	0.003	1.14	0.80–1.62	0.481†	0.080
*Genotype methods*																				
PCR-RFLP (n = 4)	1.14	0.78–1.68	0.499†	0.002	1.20	0.93–1.55	0.168	0.151	1.36	0.57–3.21	0.489†	<0.001	1.47	0.61–3.57	0.391†	0.001	1.28	0.57–2.90	0.549†	0.001
TaqMan (n = 3)	0.90	0.78–1.04	0.144†	0.376	0.90	0.73–1.12	0.343	0.385	0.88	0.70–1.09	0.244	0.744	0.82	0.61–1.09	0.162	0.398	0.90	0.71–1.14	0.401	0.956
PCR-SSCP (n = 3)	1.31	0.90–1.92	0.162†	0.032	1.30	0.92–1.83	0.136	0.279	1.49	0.83–2.70	0.184†	0.030	1.68	0.85–3.34	0.139†	0.061	1.41	0.81–2.44	0.222†	0.070
direct DNA sequencing (n = 1)	0.99	0.71–1.36	0.931	-	0.94	0.55–1.59	0.809	-	1.03	0.58–1.84	0.914	-	0.98	0.49–1.95	0.945	-	1.06	0.58–1.93	0.854	-

OR  =  odds ratios;

95%CI  =  95% confidence interval;

*P_h_*  =  *P* value of heterogeneity test;

† =  estimates for random effects model.

### Association between ESR1 XbaI (A>G) polymorphism and prostate cancer risk

**Figure 4:**
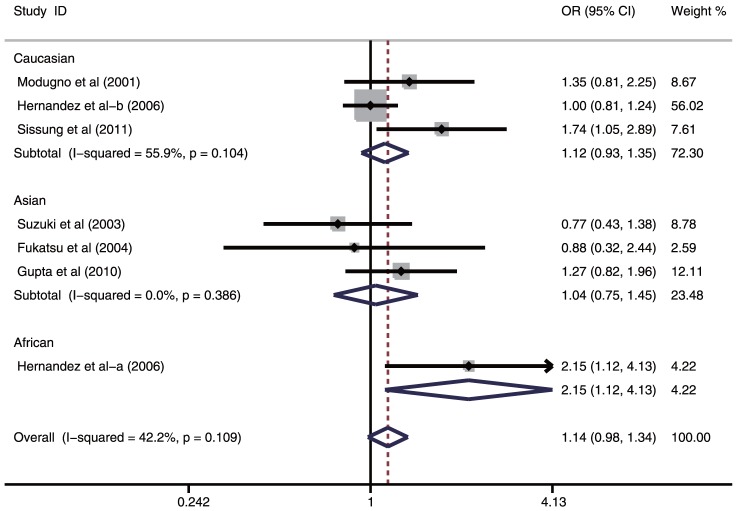
Subgroup analysis by ethnicity of ORs with a random-effects model for associations between ESR1 XbaI (A>G) polymorphism and prostate cancer risk under dominant model (GG + AG vs. AA).

As shown in Table 3, the findings of this meta-analysis on the correlation between ESR1 XbaI (A>G) and prostate cancer risk are summarized. The associations between ESR1 XbaI (A>G) polymorphism and prostate cancer risk were investigated in six studies. The heterogeneity was not obvious under all genetic models (all *P*>0.05), so fixed effects model was used. No associations were found between ESR1 XbaI (A>G) polymorphism and prostate cancer risk under any genetic models (G allele vs. A allele: OR = 1.09, 95%CI: 0.98–1.22, *P* = 0.118; GG + AG vs. AA: OR = 1.14, 95%CI: 0.98–1.34, *P* = 0.089; GG vs. AA + AG: OR = 1.08, 95%CI: 0.86–1.34, *P* = 0.523; GG vs. AA: OR = 1.19, 95%CI: 0.92–1.55, *P* = 0.174; GG vs. AG: OR = 1.03, 95%CI: 0.82–1.30, *P* = 0.797; respectively). In the subgroup analysis based on ethnicity, the results indicated that ESR1 XbaI (A>G) polymorphism might significantly increase the risk of prostate cancer among African populations (G allele vs. A allele: OR = 1.60, 95%CI: 1.00–2.57, *P* = 0.049; GG + AG vs. AA: OR = 2.15, 95%CI: 1.12–4.13, *P* = 0.022; respectively), but not enough reliability was established due to the estimation of effect size from a single study [26]. Nevertheless, ESR1 XbaI (A>G) polymorphism did not show any statistical association with the risk of prostate cancer among Caucasian and Asian populations (all *P*>0.05) (Figure 4). Results from the subgroup analysis by country showed that ESR1 XbaI (A>G) polymorphism was slightly correlated with increased risk of prostate cancer among American population under the allele model (G allele vs. A allele: OR = 1.14, 95%CI: 1.00–1.30, *P* = 0.045), but not among Japanese and Indian populations. We also performed stratified analyses based on source of controls and genotype methods. The pooled analyses showed that ESR1 XbaI (A>G) polymorphism might be associated with increased risk of prostate cancer in population-based and PCR-RFLP subgroups. However, similar associations were not found in hospital-based and Taqman or PCR-SSCP subgroups (as shown in Table 3).

**Table 3:** Meta-analysis of the association between ESR1 XbaI (A>G) polymorphism and prostate cancer risk.

Subgroups (sample sizes)	G allele vs. A allele(allele model)				GG + AG vs. AA(dominant model)				GG vs. AA + AG(recessive model)				GG vs. AA(homozygous model)				GG vs. AG(heterozygous model)			
	OR	95%CI	*P*	*P_h_*	OR	95%CI	*P*	*P_h_*	OR	95%CI	*P*	*P_h_*	OR	95%CI	*P*	*P_h_*	OR	95%CI	*P*	*P_h_*
Overall (n = 6)	1.09	0.98–1.22	0.118	0.089	1.14	0.98–1.34	0.089	0.109	1.08	0.86–1.34	0.523	0.472	1.19	0.92–1.55	0.174	0.332	1.03	0.82–1.30	0.797	0693
*Ethnicity*																				
Caucasians (n = 3)	1.11	0.97–1.27	0.125	0.099	1.12	0.93–1.35	0.229	0.104	1.21	0.92–1.60	0.177	0.251	1.26	0.94–1.69	0.127	0.120	1.17	0.87–1.58	0.291	0.397
Asians (n = 7)	0.96	0.77–1.21	0.736	0.286	1.04	0.75–1.45	0.807	0.386	0.85	0.58–1.24	0.392	0.718	0.89	0.50–1.58	0.688	0.590	0.84	0.56–1.26	0.399	0.914
Africans (n = 1)	1.60	1.00–2.57	0.049	-	2.15	1.12–4.13	0.022	-	1.22	0.43–3.44	0.713	-	1.81	0.60–5.49	0.294	-	0.81	0.27–2.40	0.704	-
*Country*																				
American (n = 3)	1.14	1.00–1.30	0.045	0.079	1.18	0.99–1.40	0.072†	0.044	1.21	0.93–1.59	0.162	0.429	1.29	0.97–1.71	0.084	0.201	1.14	0.86–1.52	0.361	0.521
Japanese (n = 2)	0.82	0.61–1.11	0.200	0.688	0.80	0.48–1.32	0.381	0.830	0.80	0.52–1.22	0.292	0.610	0.70	0.33–1.50	0.361	0.643	0.81	0.52–1.28	0.368	0.784
Indian (n = 1)	1.17	0.84–1.64	0.362	-	1.27	0.82–1.96	0.282	-	1.09	0.46–2.59	0.847	-	1.23	0.50–2.99	0.655	-	0.96	0.39–2.35	0.929	-
*Source of controls*																				
Population-based (n = 3)	1.30	1.06–1.59	0.014	0.437	1.42	1.08–1.88	0.013	0.635	1.38	0.86–2.20	0.179	0.302	1.64	1.00–2.68	0.050	0.252	1.17	0.72–1.91	0.529	0.366
Hospital-based (n = 3)	1.02	0.89–1.17	0.794	0.127	1.04	0.86–1.25	0.721	0.113	1.00	0.78–1.29	0.992	0.578	1.06	0.79–1.44	0.688	0.523	0.99	0.76–1.29	0.962	0.664
*Genotype methods*																				
PCR-RFLP (n = 2)	1.34	1.05–72	0.019	0.238	1.45	1.04–2.02	0.027	0.354	1.64	0.90–2.99	0.110	0.204	1.94	1.04–3.64	0.039	0.155	1.39	0.74–2.60	0.300	0.261
TaqMan (n = 1)	1.07	0.92–1.25	0.357	0.078	1.08	0.88–1.32	0.461	0.029	1.31	0.83–1.54	0.436	0.887	1.15	0.83–1.60	0.404	0.405	1.10	0.80–1.53	0.554	0.557
PCR-SSCP (n = 3)	0.95	0.75–1.21	0.679	0.289	1.04	0.73–1.48	0.834	0.345	1.64	0.90–2.99	0.383	0.740	0.91	0.52–1.58	0.725	0.570	0.83	0.56–1.23	0.346	0.952

OR  =  odds ratios;

95%CI = 95% confidence interval;

Ph  =  P value of heterogeneity test;

† =  estimates for random effects model.

### Association between other SNPs in ESR1 gene and prostate cancer risk

Moreover, we also performed a pooled analysis for all eligible case-control studies to explore the role of ESR1 codon 10 (T>C), codon 325 (C>G), codon 594 (G>A) and +261G>C polymorphisms in prostate cancer susceptibility. However, no significant association between these SNPs and the risk of prostate cancer was observed (all *P*<0.05) (as shown in Supplement S4).

### Sensitivity analysis

Sensitivity analysis was performed to assess the influence of each individual study on the pooled ORs by omission of individual studies. The analysis results suggested that no individual studies significantly affected the pooled ORs in both ESR1 PvuII (C>T) and XbaI (A>G) polymorphisms under the dominant model (as shown in Supplement S5). In addition, we also performed a sensitivity analysis by excluding four studies that deviated significantly from HWE. Further analysis showed that these four non-HWE studies also have no effects on the pooled ORs in both ESR1 PvuII (C>T) and XbaI (A>G) polymorphisms under the dominant model (as shown in Supplement S6).

### Publication bias

**Figure 5:**
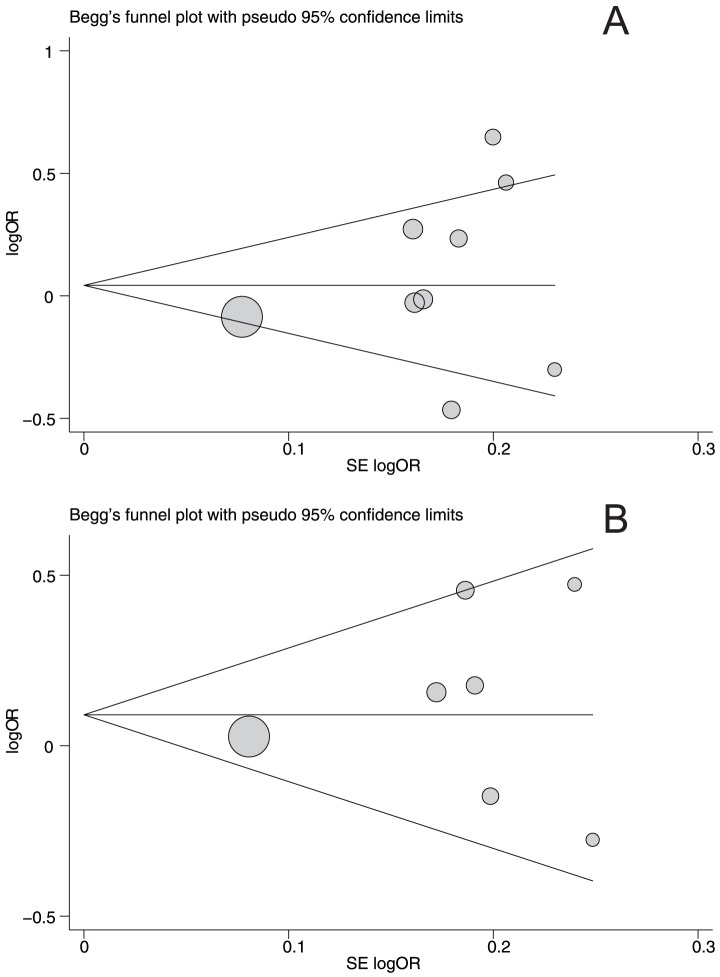
Begger's funnel plot of the meta-analysis of ESR1 PvuII (A) and XbaI (B) polymorphisms with prostate cancer risk under dominant model. Each point represents a separate study for the indicated association. Log[OR], natural logarithm of OR. Horizontal line, mean magnitude of the effect. Note: Funnel plot with pseudo 95% confidence limits was used.

Begger's funnel plot and Egger's linear regression test were performed to assess the publication bias of included studies. The shapes of the funnel plots did not reveal any evidence of obvious asymmetry under the dominant model (Figure 5). Egger's test also did not show any significantly statistical evidence of publication bias under the dominant model (PvuII: *t* = 0.88, *P* = 0.399; XbaI: *t* = 1.03, *P* = 0.350).

## Discussion

Estrogen plays an important role in the expression of genes that regulate hormone levels, normal prostate developments and prostate diseases [24], [26]. Aberrant expressions or mutations of hormone receptors in cancer cells were also found to be associated with prostate cancer aggressiveness [34]. Additionally, inherited variants in sex hormonal receptor genes may perhaps interact with other variants in the steroidogenic and metabolic pathways cooperatively [5]. Therefore, hormonal status is clearly an important factor in prostate cancer biology. Estrogen exerts its effects on prostatic tissues by binding to and activating estrogen receptors (ESR1 and ESR2). Estrogen receptor (ESR1) is involved in sex steroid metabolism and functions in carrying out the proper cellular responses [27]. Accumulating evidences also indicate that estrogen and estrogen receptors play crucial roles in prostate cancer development and progression [33]. ESR1 is expressed in prostate stromal cells and is thought to stimulate growth factor release and cause epithelial cell proliferation. Ricke et al suggested that it is likely an imbalance of their expression may be critical in determining the effects that estrogen ultimately has on prostate cancer cells [35]. However, a recent genetic study showed that mutations in ESR1 were independent risk factors [28].

Human ESR1 encoding gene is located on chromosome 6q24–27, consists of eight exons and seven introns, and is about 140 kb in length with two promoter regions and five functional domains, designated as A/B–F, in two differing transcripts at the 5′ region [30]. The protein itself has 595 amino acids and weights a molecular weight of 66,182 Da [4]. In the normal prostate, ESR1 is expressed in stromal cells but not in epithelial cells. In contrast, it has been discovered that ESR1 is expressed in the epithelium in malignant prostate tissues [38], [39]. ESR1 gene mutations may alter the concentration of reactive estrogens in the prostate [28]. Several polymorphisms in ESR1 gene, such as PvuII (rs9340799 A>G) and XbaI (rs2234693 C>T), have been studied to a assess their causal relationships with prostate cancer [23], [25], [37], [40]. It appears that inherited alterations of the ESR1 gene can possibly explain interpopulation differences in the incidences associated with estrogen-related diseases [29]. Many investigations have demonstrated that prostate cancer risk was associated with the ESR1 gene polymorphism [36], [37].

To explore the association between ESR1 gene polymorphisms and prostate cancer risk, we performed a meta-analysis on 2,165 prostate cancer cases and 3,361 controls. This is the first meta-analysis exploring the relationship between prostate cancer and the ESR1 gene polymorphisms. When all the eligible studies were pooled into the meta-analysis, the results showed that ESR1 PvuII (C>T) and XbaI (A>G) polymorphisms were not associated with the risk of prostate cancer, yet many studies have inferred that ESR1 gene polymorphisms were related to the onset and develop of prostate cancer [11], [12], [26], [37], [41]–[43]. A possible reason for the controversy is that a considerable degree of heterogeneity existed among the other studies due to differences in sample sizes, exposure estimates, ethnicity, source of controls and other potential confounding variables. Therefore, we performed a stratified analysis based on ethnicity and country. The results showed that ESR1 PvuII (C>T) polymorphism might increase the risk of prostate cancer among Asian populations, especially among Indian population. ESR1 XbaI (A>G) polymorphism was confirmed to be associated with increased risk of prostate cancer among American population under the allele model, but not among Japanese and Indian populations. However, pooled estimates for Indian population was slightly higher than that for American population, and only pooled OR under the allele model was significant and might lead to unacceptably low levels of statistical power. Therefore, this result should be verified by large, well-designed epidemiologic population-based studies. Ethnic differences in prostate cancer susceptibility are probably the results of both genetic and epidemiological factors, which may mainly be the results of genetic factors including mutations in rare genes that confer high risks and/or mutations in specific genes that confer modestly increased risks [44]. Furthermore, we also performed a pooled analysis for all eligible case-control studies to explore the role of codon 10 (T>C), codon 325 (C>G), codon 594 (G>A) and +261G>C polymorphisms in prostate cancer risk. However, no significant associations between these SNPs and prostate cancer risk were observed.

In interpreting the results of the current meta-analysis, some limitations need to be addressed. First, the sample size is still relatively small and may not provide sufficient power to estimate the association between ESR1 gene polymorphisms and prostate cancer risk. Second, heterogeneity across studies was obvious, which might be a result of the difference in ethnicity, country, source of controls and genotype methods. Third, the selection bias may exist because only articles published in English or Chinese were included. Besides, our meta-analysis was also based on unadjusted ORs estimates because not all published studies presented adjusted ORs, or when they were, the ORs were not adjusted by the same potential confounders, such as ethnicity, age, gender, geographic distribution, etc. Although no obvious publication bias was identified, potential bias cannot be completely ruled out. Nonetheless, it is well acknowledged that many other factors, such as gene-gene or gene-environment interactions may affect the risk of gastrointestinal cancer. Finally, although all cases and controls of each study were well defined with similar inclusion criteria, there may be potential factors that were not taken into account that could have influenced our results.

In spite of these limitations, our meta-analysis still had some merits and values. To the best of our knowledge, this is the first meta-analysis on the relationship of ESR1 gene polymorphisms and prostate cancer risk. It is worthwhile to mention that we also established an efficient searching strategy based on computer-assisted programs and manual searches, which allowed us to include as many studies as possible. According to our selection criteria, the quality of studies included in this meta-analysis is sufficient. Explicit methods for study selection, data extraction, and data analysis were well designed before initiating. Finally, there was no evidence of publication bias in this meta-analysis and the sensitivity analysis indicated that the results are statistically robust.

In summary, this meta-analysis suggested that ESR1 PvuII (C>T) polymorphism may be a potential risk factor for prostate cancer among Asian populations, especially among Indian population; while ESR1 XbaI (A>G) polymorphism may increase the risk of prostate cancer among American population. Such relationship can provide a more comprehensive mechanism of how ESR1 mutations function in the development of prostate cancer, as well as promise a more effective treatment for prostate cancer. However, further studies are still needed to warrant and validate the association between ESR1 gene polymorphism with other genetic polymorphisms and prostate cancer risk.

## Supporting Information

Supplement S1 **PRISMA Checklist.**
(DOC)

Supplement S2 **Modified STROBE quality score systems.**
(DOC)

Supplement S3 **The Newcastle-Ottawa Scale for assessing methodological quality of case-control studies.**
(DOC)

Supplement S4 **Meta-analysis of the association between other four SNPs of ESR1 gene and prostate cancer risk.**
(DOC)

Supplement S5 **Sensitivity analysis of the summary odds ratio coefficients on the association of ESR1 PvuII (C>T) and XbaI (A>G) polymorphisms with prostate cancer risk under dominant model.**
(EPS)

Supplement S6 **Sensitivity analysis after exclusion of four studies deviating from HWE on the association of ESR1 PvuII (C>T) and XbaI (A>G) polymorphisms with prostate cancer risk under dominant model.**
(EPS)
