# Supplementary material for: ESR1 Gene Polymorphisms and Prostate Cancer Risk: A HuGE Review and Meta-Analysis
Source: PLoS One. 2013 Jun 21;8(6):e66999. doi: 10.1371/journal.pone.0066999 (PMC3689664; doi:10.1371/journal.pone.0066999)
Supplement: Supplement S4 — Meta-analysis of the association between other four SNPs of ESR1 gene and prostate cancer risk. (DOC) [file pone.0066999.s004.doc]

**Supplement S4 Meta-analysis of the association between other four SNPs in ESR1 gene and prostate cancer risk.**

| SNPs (sample sizes) | 2 allele vs 1 allele  (allele model) | | | | 1/2 + 2/2 vs 1/1  (dominant model) | | | | 2/2 vs 1/1 + 1/2  (recessive model) | | | | 2/2 vs 1/1  (homozygous model) | | | | 2/2 vs 1/2  (heterozygous model) | | | |
| --- | --- | --- | --- | --- | --- | --- | --- | --- | --- | --- | --- | --- | --- | --- | --- | --- | --- | --- | --- | --- |
|  | OR | 95%CI | *P* | *P_h_* | OR | 95%CI | *P* | *P_h_* | OR | 95%CI | *P* | *P_h_* | OR | 95%CI | *P* | *P_h_* | OR | 95%CI | *P* | *P_h_* |
| Codon 10 (T>C) (n = 4) | 1.07 | 0.72-1.61 | 0.731^†^ | 0.007 | 1.16 | 0.76-1.76 | 0.487^†^ | 0.032 | 1.10 | 0.64-1.91 | 0.724^†^ | 0.058 | 1.22 | 0.49-3.00 | 0.669^†^ | 0.004 | 1.04 | 0.76-1.42 | 0.804 | 0.266 |
| Codon 325 (C>G) (n = 4) | 1.04 | 0.85-1.27 | 0.700 | 0.252 | 1.02 | 0.82-1.28 | 0.854 | 0.657 | 1.03 | 0.68-1.57 | 0.891 | 0.182 | 1.09 | 0.67-1.75 | 0.732 | 0.171 | 1.05 | 0.67-1.65 | 0.838 | 0.286 |
| Codon 594 (G>A) (n = 3) | 1.16 | 0.96-1.40 | 0.133 | 0.506 | 1.18 | 0.92-1.52 | 0.201 | 0.459 | 1.24 | 0.85-1.81 | 0.268 | 0.891 | 1.26 | 0.84-1.90 | 0.265 | 0.812 | 1.20 | 0.80-1.80 | 0.377 | 0.982 |
| +261G>C (n = 2) | 0.95 | 0.67-1.37 | 0.797 | 0.563 | 0.97 | 0.66-1.42 | 0.869 | 0.578 | 0.69 | 0.13-3.58 | 0.657 | - | 0.68 | 0.13-3.54 | 0.647 | - | 0.73 | 0.14-3.97 | 0.717 | - |

OR = odds ratios; 95%CI = 95% confidence interval; 1 = wild allele; 2 = mutant allele; 1/1 = wild homozygote; 1/2 = heterozygote; 2/2 = mutant homozygote; *P_h_* = *P* value of heterogeneity test; † = estimates for random effects model.
